# Effect of Dietary Supplementation with a Natural Extract of *Sclerocarya birrea* on Glycemic Metabolism in Subjects with Prediabetes: A Randomized Double-Blind Placebo-Controlled Study

**DOI:** 10.3390/nu13061948

**Published:** 2021-06-06

**Authors:** Desirée Victoria-Montesinos, Maravillas Sánchez-Macarro, José Antonio Gabaldón-Hernández, María Salud Abellán-Ruiz, María Querol-Calderón, Antonio J. Luque-Rubia, Enrique Bernal-Morell, Vicente Ávila-Gandía, Francisco Javier López-Román

**Affiliations:** 1 Health Sciences Department, Campus de los Jerónimos, Universidad Católica San Antonio de Murcia (UCAM), Carretera de Guadalupe s/n, 30107 Murcia, Spain; dvictoria@ucam.edu (D.V.-M.); msanchez4@ucam.edu (M.S.-M.); jagabaldon@ucam.edu (J.A.G.-H.); msabellan@ucam.edu (M.S.A.-R.); mquerol@ucam.edu (M.Q.-C.); ajluque@ucam.edu (A.J.L.-R.); vavila@ucam.edu (V.Á.-G.); 2 Unit of Infectious Diseases, Hospital General Universitario Reina Sofía, 30003 Murcia, Spain; ebm.hgurs@gmail.com; 3 Primary Care Research Group, Biomedical Research Institute of Murcia (IMIB-Arrixaca), 30120 Murcia, Spain

**Keywords:** prediabetes, *Sclerocarya birrea*, glucose metabolism, insulin resistance, lipid profile, nutraceutical product, clinical trial

## Abstract

A randomized, double-blind, placebo-controlled study was conducted with the primary objective of assessing the effect of a natural extract of *Sclerocarya birrea* on glucose metabolism in subjects with prediabetes. The duration of the study was 90 days. Thirty-three subjects assigned to the experimental group (daily ingestion of 100 mg of the nutraceutical product) and 34 assigned to the placebo group completed the study. There were 36 men and 31 women with a mean age of 32.3 ± 14.1 years. In the area under the curve (AUC) of the oral glucose tolerance test (OGTT), statistically significant decreases in the experimental group at 40 and 90 days as compared with baseline were found, whereas significant changes in the placebo group were not observed. Within-group differences were statistically significant in favor of the experimental group for glucose peak at OGTT, serum insulin, insulin resistance markers, and flow-mediated dilation. Changes in lipid and anthropometric parameters were not observed, although there was a trend for lower cholesterol levels and a decrease in body weight in the experimental group. Decreases in systolic blood pressure were also higher among subjects in the experimental group. This exploratory study confirms the antidiabetic activity of *Sclerocarya birrea* in prediabetes. Further studies using better measurements of beta-cell function are needed to clarify the underlying mechanisms of the hypoglycemic effect of this natural compound.

## 1. Introduction

Prediabetes is an intermediate state of hyperglycemia with glycemic parameters above normal but below the diabetes threshold. The specific parameters included in the criteria for the definition of prediabetes are not uniform, with impaired glucose tolerance (IGT) of 140–199 mg/dL after ingestion of 75 g of oral glucose load, impaired fasting glucose (IFG) of 100 or 110 to 125 mg/dL, and an additional criterion of hemoglobin A1c (HbA1c) at a level of 5.7% to 6.4% [1,2]. According to the American Diabetes Association (ADA), relying on one test may underestimate the prevalence of prediabetes [1].

Prediabetes is increasingly recognized as an important metabolic state, predisposing individuals to progression to overt type 2 diabetes (25% of subjects within 3–5 years and as many as 70% within their lifetime) [3] and diabetes-associated complications, such as diabetic retinopathy, neuropathy, nephropathy, and cardiovascular disease [4,5]. The 2019 Ninth Edition of the International Diabetes Federation (IDF) atlas reported an estimated cumulative incidence of type 2 diabetes progression 5 years after diagnosis of IGT or IFG between 26% and 50%, respectively [6]. Moreover, the estimated number of adults aged 20–79 years with IGT was 7.5% of the world population in this age group (374 million), predicted to rise to 8.0% (454 million) by 2030 and to 8.6% (548 million) by 2045 [6]. These estimates are based on IGT only, so the prevalence of prediabetes may be higher if additional criteria are taken into consideration [7].

Insulin resistance and defective glucose sensing at the pancreatic *β*-cell are the central pathophysiologic determinants that together cause hyperglycemia. The mechanisms involved include deficient muscle gluconeogenesis, oxidative stress and increased reactive oxygen species (ROS) [8], defective expression or translocation of glucose transporter 4 (GLUT4) [9], imbalance of the phosphatidylinositol 3-kinase (PI3K)/protein kinase B (AKT) (PI3K/AKT) signaling pathway [10], endoplasmic reticulum (ER) stress with activation of unfolded protein response, and activation of the c-Jun N-terminal kinase pathway [11]. On the other hand, prediabetes is considered an underlying etiology of metabolic syndrome [12].

Lifestyle interventions are a very important approach in the management of prediabetes. Changing the modifiable risk factors by targeting obesity with increased physical activity and dietary changes improves insulin sensitivity and preserves *β*-cell function [13,14]. Interventions using naturally occurring molecules are a focus of increasing attention as complementary nutritional strategies to ameliorate insulin resistance, glycemic control, and lipid profile. A variety of nutraceutical supplements have been used in clinical practice to ameliorate the underlying mechanisms of insulin resistance, including L-carnitine, *α*-lipoic acid, berberine, omega-3 polyunsaturated fatty acids, and phytochemicals including polyphenols, flavonoids, and phenolic acids [15,16].

*Sclerocarya birrea* (A. Rich.) *Hochst* belonging to the Anacardiaceae family (known as marula) is a medium-sized deciduous African tree, where leaves, stem bark, root, and fruits have a wide range of uses in food and traditional medicine. Chemical constituents include ascorbic acid, sesquiterpene hydrocarbons, amino acids, oleic, myristic and stearic fatty acids, tannins, flavonoids, and polyphenols, among others, which have been the basis of biological studies assessing the antioxidant, anti-inflammatory, analgesic, antiparasitic and antimicrobial activity, as well as antidiabetic properties of *Sclerocarya birrea* [17]. A number of studies in experimental animals have shown the hypoglycemic effect and antidiabetic activity of *Sclerocarya birrea* [18,19,20,21]. These studies showed how *Sclerocarya birrea* might contribute to different metabolic processes, with a possible mechanism of action similar to α-glycosidases and α-amylases [18], being involved similarly to sulfonylurea potentiating calcium signaling in beta-cells [19], or enhancing AMPK activation through a mechanism similar to that of biguanides [20]. However, results about human intervention trials were not found in the literature.

Regarding the target pathology, *Sclerocarya birrea* is used as a traditional remedy for diabetes mellitus from ancient times. In a survey conducted between January and April 2016, a total of 116 diabetic patients distributed in 58 tribes and in all phytogeographic units, previously diagnosed in the hospital of Cameroon, responded to the questionnaire about the use of medicinal plants to treat their disease. Among them, *Sclerocarya birrea* was used by 48% of surveyed people. The treatment consisted of boiling 250 g of stem-bark or 100 g of leaves in 4 L of water and drinking 250 mL of the decoction three times per day [22].

Due to the traditional use of the source *Sclerocarya birrea* in the ethnopharmacological treatment of diabetes, the objective of the present study was to assess the efficacy of an aqueous extract obtained from the bark of the *Sclerocarya birrea* tree in the control of glucose metabolism in subjects with prediabetes as compared with placebo. As far as we are aware, this is the first exploratory clinical trial conducted in clinical practice to assess the effect of a nutraceutical compound based on *Sclerocarya birrea* in subjects with a confirmed diagnosis of prediabetes.

## 2. Materials and Methods

### 2.1. Study Design and Participants

This was a single-center, randomized, double-blind, placebo-controlled study with two parallel arms stratified by sex, conducted at the Health Sciences Department of Universidad Católica San Antonio de Murcia (UCAM), in Murcia, Spain. The study began on 1 July 2018 and finished on 13 December 2019. The primary objective of this exploratory study was to assess the effect of a 90-day daily regimen with a dietary nutraceutical supplement based on a natural extract of *Sclerocarya birrea* on the control of glucose metabolism in subjects diagnosed with prediabetes. Secondary objectives included changes in the lipid profile, endothelial function, inflammatory biomarkers, anthropometric variables, and assessment of the tolerability and safety of the study product. Participants were mainly recruited by advertising the study through mass media and talks in women’s centers, elderly community centers, and neighborhood associations. Eligible subjects were Caucasian men and women aged between 18 and 65 years, diagnosed with prediabetes according to ADA criteria [1] (HbA1c level of 5.7% to 6.4%, fasting plasma glucose (FPG) 100–125 mg/dL, oral glucose tolerance test (OGTT) 140–199 mg/dL), body mass index (BMI) between 20 and 35 kg/m^2^, and stable nutritional habits with no body weight gain or loss of more than 5 kg in the last 10 weeks. Exclusion criteria were history of liver or renal dysfunction, alcohol consumption (>20 g/day), use of any drug that may affect glucose metabolism, participation in another clinical trial within the previous 3 months, pregnancy, and ineligibility as judged by the investigators.

The study protocol was approved by the Ethics Committee of Universidad Católica San Antonio (code 6113, approval date 3 June 2016) (Murcia, Spain) and was registered in the ClinicalTrials.gov, accessed on 31 October 2019 (NCT04132908). Written informed consent was obtained from all participants.

### 2.2. Intervention and Study Procedures

Participants were randomly assigned to the intervention group (dietary intervention with the *Sclerocarya birrea*-based nutritional supplement) or the control group (supplementation with placebo) using the Epidat 3.1 program but ensuring the homogeneity of the study groups regarding sex. Randomization was performed by an independent researcher.

The active product was an encapsulated dry powder aqueous extract obtained from *Sclerocarya birrea* stem bark. The product (Sclerobigenol^®^) was supplied by Herbafor, S.L. (Fortuna, Murcia, Spain) and was manufactured by a combination of physical stages, including air drying of the raw strands of the bark tree after being collected, the grinding of the dry bark to an adequate particle size, an aqueous maceration at ambient temperature for a fixed period of time, the separation of solid matter from the solution by successive filtrations, and the final elimination of water from the eluted solution by steam-distillation and subsequent drying under mild temperature and high vacuum. The resultant product is then sampled for analysis, filled into aseptic bags, labeled, and packed in cardboard boxes until use.

In order to determine the phenolic composition of the final product, a comprehensive characterization of the extract was carried out by high−performance liquid chromatography coupled to electrospray time of flight mass spectrometry (HPLC−ESI−TOF/MS), as described by Jiménez-Sánchez et al. [23]. The quantification of 14 major phenols contained in the extract was also investigated.

**Figure 1:**
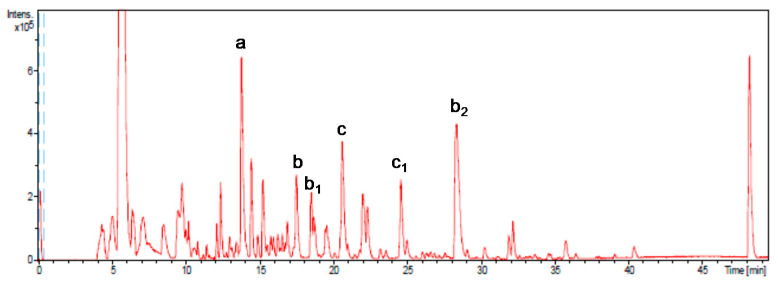
Base peak chromatogram obtained by HPLC-SI-TOF/ MS. More representative peaks such as gallic acid (a), epigallocatechin gallate (b), isomers (b_1_ and b_2_), procyanidin B2-3,3-di-O-gallate (c), and isomer (c_1_) have been identified.

The major compound identified (Figure 1) was gallic acid (compound a; 1588 μg/g on average), constituting 45% of the total phenolic fraction quantified. Other major representative compounds were epigallocatechin gallate (compound b and isomers b1 and b2, 593 μg/g on average), representing 17% of the total, and procyanidin B2 di-O-gallate (compound c and isomer c1, 511 μg/g on average), with 15% of the total.

The stability of the powder extract in capsule formulation was investigated for a period of 90 days, under accelerated conditions (stored at 40 °C and 45% humidity), by total phenolic compounds determination and quantitative analysis of more representative compounds of the phenolic profile, according to Jiménez-Sánchez et al. (2015) [23].

The analysis of the total phenolic compounds in the capsules by the Folin–Ciocalteu method remained unchanging after 90 days of storage in accelerated conditions. In general, catechin provided losses during storage, but the increase of gallic and epicatechin levels balanced the catechin loss, maintaining the global phenolics variations of the compounds. Therefore, it can be considered that the capsules show normal stability behavior during storage under accelerated conditions when using phenolic compounds as reference stability markers.

The qualitative composition of the product includes a variety of 95 components, including organic acids, polyphenols, fatty acids derivatives, etc., being that polyphenols, especially dimers of epicatechins, epigallocatechins, epigallocatechin gallate, procyanidin B2- 3,3-di-O-gallate, and organic acids such as gallic acid are the most relevant. All participants followed the same regimen of taking two capsules/day of the study product at the time of breakfast and dinner for 90 days. Subjects who were assigned to the experimental group received the active product (2 capsules/day, 2 × 50 mg each), and those assigned to the control group received identical-appearing placebo capsules (maltodextrin).

Participants were visited at baseline (visit 1) and at 40 (visit 2) and 90 days (final visit). At the baseline visit, the written informed consent was obtained, and fulfillment of the inclusion criteria was checked. The study product was provided at visits 1 and 2. Clinical assessments at each study visit included detailed medical history, measurement of glucose metabolism variables, lipid profile, anthropometric variables, blood pressure, brachial artery plethysmography (performed at baseline and at the final visit), and biomarkers of inflammation. Venous blood samples were taken after 12 h fasting at each of the visits for laboratory analysis. Participants were advised against performing moderate or severe physical exercise for at least 1 day before blood sampling. Alterations in dietary habits and the use of other nutritional supplements were not allowed during the study. The returned capsules at visits 2 and 3 (end of the study) were checked and counted for adherence with the study product. Participants were directly asked for adverse events, and results of laboratory analyses were assessed for normality.

### 2.3. Study Variables

Glucose metabolism variables included the area under the curve (AUC) of OGTT (2 h OGTT after 12 h overnight fasting, 75 g glucose, capillary blood samples obtained at 0, 15, 30, 45, 60, 90, and 120 min), which was calculated based on the AUC at fasting serum glucose and based on the X-axis at glucose concentration of 0 mg/dL (AUC at 0 mg/dL), using the trapezoidal method for both variables: 1 h and 2 h post-OGTT glucose levels, serum HbA1c by high-performance liquid chromatography, fasting serum glucose (clinical chemistry analyzer B400 ByoSystems) and insulin levels, insulin/glucose ratio, homeostatic model assessment: insulin resistance (HOMA-IR) with a cut-off of >3.2 for diagnosis of IR; HOMA-β1, the Quantitative Insulin Sensitivity Check Index (QUICKI) with a cut-off of <0.35 for diagnosis of IR, lipid profile (cholesterol, low-density lipoprotein cholesterol (LDL-C), high-density lipoprotein cholesterol (HDL-C) and triglycerides) (B400 ByoSystems), blood pressure (BP), flow-mediated dilation (FMD) as a measure of endothelial function, anthropometric variables (body weight, BMI, hip circumference, waist-to-hip ratio, and free fat mass), and inflammation-related biomarkers (E-selectin, interleukin-6 (IL-6)).

Blood pressure was measured coinciding with blood withdrawal in the morning using an OMRON M6 AC blood pressure monitor (Omron Healthcare España).

The measurement of blood pressure was performed with an OMRON M6 AC blood pressure monitor (Omron Healthcare España) at the time of peripheral blood sampling. Blood pressure was recorded after participants were comfortably seated in a quiet room for 5 min. Blood pressure values were the average of three measurements taken 2 min apart [24]. FMD was measured following the recommendations of the International Brachial Reactivity Task Force [25] in the brachial artery. We used high-resolution echo-Doppler ultrasound equipment (SonoSite MicroMaxx HFL38) with a 6–13 MHz linear array transducer. After placing a blood pressure sphygmomanometer cuff above the antecubital fossa, the brachial artery diameter was measured following the acquisition of a baseline resting image. The cuff was then inflated up to suprasystolic pressure for 5 min in order to provoke arterial occlusion, and a new measurement was obtained after 1 min of deflating the cuff. FMD was determined using the equation: (peak of hyperemia diameter) – (baseline diameter)/(baseline diameter) × 100, with results after occlusion expressed as the percentage of arterial diameter change (mm) in response to hyperemia as compared to the baseline diameter. All measurements were taken by the same investigator. Bio-impedance analysis (BIA) was used to determine free fat mass using a whole-body BIA analyzer Tanita BC-420MA (Tanita Corporation, Arlington Heights, IL, USA). Serum levels of E-selectin and IL-6 were measured by E-selectin and IL-6 High Sensitivity ELISA kits (IBL International, GmbH, Hamburg, Germany).

Safety variables were assessed in the following laboratory tests: complete hemogram; liver functions tests, including bilirubin, aspartate aminotransferase (AST), alanine aminotransferase (ALT), gamma-glutamyl transpeptidase (GGT) and lactate dehydrogenase (LDH); as well as serum creatinine levels and blood urea nitrogen.

Physical activity was measured using the Global Physical Activity Questionnaire (GPAQ) [26], and participants were classified into four categories: inactive (1), moderately inactive (2), moderately active (3), and very active (4).

### 2.4. Statistical Analysis

The sample size was calculated according to the AUC during OTTG as the main variable of the study. Considering a standard deviation of AUC during OTTG of 786 mg·min/dL reported in a similar population [27], for a precision of 500 mg·min/dL with an alpha risk of 5% and statistical power of 80%, 32 subjects in each group were needed, increasing to 35 subjects per group assuming a 10% loss to follow-up.

The analysis was performed by a non-blinded statistician according to the double-blind design of the study and was based on the per-protocol (PP) data set corresponding to those participants who finished the study at 90 days. The AUC of OGTT was the primary outcome of the study and the other variables were secondary outcomes. Categorical variables are expressed as frequencies and percentages and continuous variables as mean ± standard deviation (SD). The chi-square (χ^2^) test or the Fisher’s exact test were used for the comparison of categorical variables between the study groups, and the analysis of variance (ANOVA) for repeated measures with two study factors: the within-subject factor (time: baseline, 40 days, and 90 days) and between-subject factor (intervention: active product and placebo) for paired data. The G*Power 3.1 program was used to calculate statistical power. The SPSS version 21.0 (IBM Corp., Armonk, NY, USA) was used for data analysis, with a *p* < 0.05 value as statistically significant.

## 3. Results

**Figure 2:**
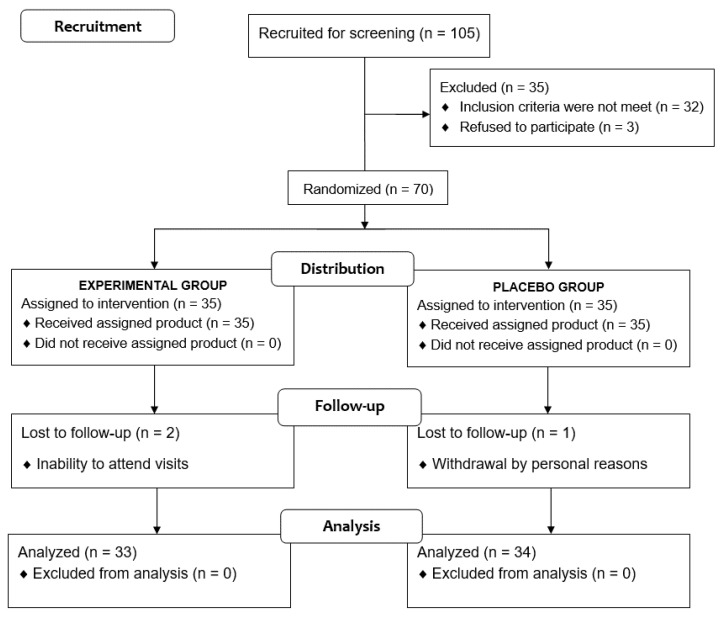
Flow chart of the study population.

Of a total of 105 voluntary subjects, 35 were excluded because the inclusion criteria were not met. The remaining 70 subjects were randomized (35 to each study group), but two subjects assigned to the experimental group and one assigned to the placebo group were lost to follow-up. Therefore, the final study population included 67 subjects (33 in the experimental group, 34 in the placebo group), 36 men and 31 women, with a mean age of 32.3 ± 14.1 years (Figure 2). Baseline characteristics of participants are shown in Table 1.

### 3.1. Glucose Metabolism

Changes in the different variables of glucose metabolism in the control and experimental groups during the study period are shown in Table 2.

**Figure 3:**
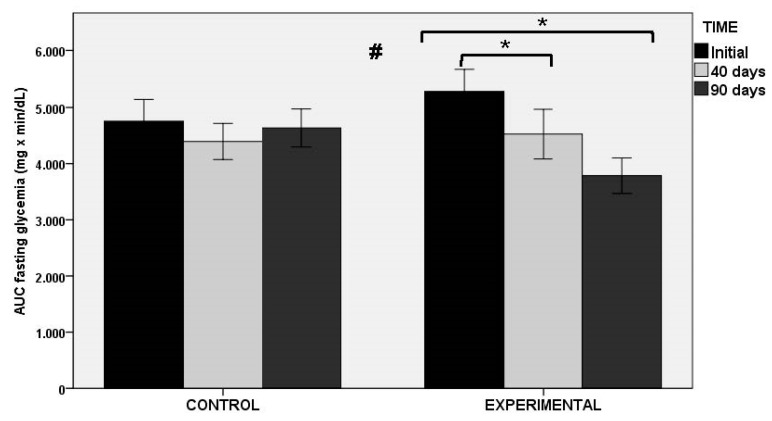
AUC of OGTT at fasting glycemia in the control and experimental groups; * *p* < 0.001 for within-group comparisons in the experimental group; # *p* < 0.004 for the comparison between control and experimental groups.

In relation to the AUC of OGTT as the primary outcome of the study (both at fasting glucose level and at min 0), statistically significant decreases in the experimental group at 40 and 90 days as compared with baseline were found, whereas significant changes in the placebo group were not observed. Between-group differences were statistically significant (Figure 3). Similar findings were observed in glucose peak levels at OGTT, with significant decreases in the experimental group at 40 and 90 days (*p* < 0.001), and between-group differences (*p* < 0.011). Changes in HbA1c and fasting serum glucose levels were not observed in any of the groups throughout the study period. However, there were differences in insulin-related markers, with statistically significant differences between the experimental and control groups for fasting serum insulin levels (*p* < 0.027), HOMA-IR (*p* < 0.034), and QUICKI index (*p* < 0.034).

### 3.2. Flow-Mediated Dilation

**Figure 4:**
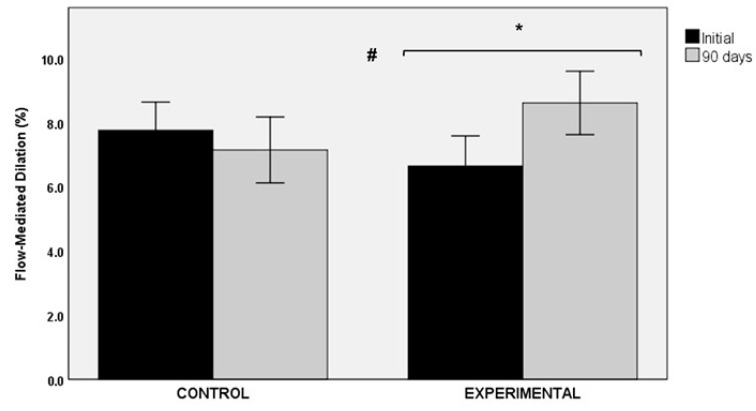
Changes in flow-mediated dilation (FMD) in the control and experimental groups; * *p* < 0.040 for within-group comparison in the experimental group; # *p* < 0.05 for the comparison between the control and the experimental groups.

The use of the nutraceutical product of *Sclerocarya birrea* over a period of 90 days was associated with an improvement in flow-mediated dilation (FMD). The comparison of baseline (6.51 ± 5.11%) and final values (8.61 ± 5.22%) showed statistically significant differences in the experimental group (*p* < 0.040, statistical power 37%). Changes in the control group were not significant (7.76 ± 5.04% vs. 7.14 ± 5.91%, *p* = 0.505, statistical power 7.4%). Between-group differences were statistically significant (*p* < 0.05) (Figure 4).

### 3.3. Lipid Profile

As shown in Table 3, statistically significant changes in the serum cholesterol, LDL-C, HDL-C, and triglycerides during the study period were not observed either in the experimental or in the control group.

### 3.4. Anthropometric Variables

Anthropometric variables did not show statistically significant changes in subjects assigned to the control or the experimental group over the 90-day study period (Table 4).

### 3.5. Inflammatory Biomarkers

Changes in inflammatory biomarkers are shown in Table 5. Mean serum levels of IL-6 were 4.37 ± 1.37 and 4.34 ± 1.79 pg/mL at baseline in the control and experimental groups, respectively, and at the end of the study, the corresponding values were 4.36 ± 1.41 and 4.08 ± 1.41 pg/mL. Within-group differences were not significant (control group, *p* = 1.0; experimental group, *p* = 0.159). Additionally, statistically significant differences in the between-group comparison were not found (*p* = 0.284).

However, serum levels of E-selectin were similar at baseline (16.62 ± 1.53 vs., 17.59 ± 1.11 ng/mL in the control and experimental groups, respectively), but showed a significant decrease in the experimental group at the end of the study (15.70 ± 1.06 ng/mL) (*p* < 0.001), whereas changes in controls were not significant (16.23 ± 0.96 ng/mL) (*p* = 0.295). Between-group differences in this variable were statistically significant (*p* < 0.001).

### 3.6. Blood Pressure

Changes in blood pressure are shown in Table 5. In the control group, the mean systolic BP (SBP) was 120 ± 18 mmHg at baseline and 120 ± 16 mmHg at the end of the study (*p* = 1.0). In the experimental group, the mean SBP was 125 ± 18 mmHg at baseline and 119 ± 17 mmHg at the end of the study (*p* < 0.001). However, significant between-group differences were not observed (*p* = 0.259). In relation to diastolic BP (DBP), differences both in the control group (baseline 76 ± 11 mmHg vs. end of study 76 ± 9 mmHg; *p* = 1.0) and in the experimental group (baseline 78 ± 10 mmHg vs. end of study 76 ± 12 mmHg; *p* = 0.265) were not significant. Additionally, between-group differences were not significant (*p* = 0.132).

In relation to physical activity, statistically significant differences between baseline and the end of the study were not observed either in the control group (*p* = 0.475) or in the experimental group (*p* = 0.127).

The study products were well tolerated and no adverse effects were recorded. All participants consumed a percentage higher than 90% of capsules.

## 4. Discussion

The administration for 90 days of a nutraceutical product based on a natural extract of *Sclerocarya birrea* in subjects diagnosed with prediabetes was associated with an improvement of the AUC during OGTT, measured using either the basal glycemia or at 0 mg/dL. Improvements were statistically significant in subjects assigned to the experimental group, whereas differences among those taking placebo (controls) were not found. As may be expected, improvements in glucose peak during OGTT were also significant in the experimental group only. These effects on glycemic control in the experimental group were also associated with decreases in insulin levels and markers of insulin resistance. Improvement of insulin resistance in the experimental group is supported by post-intervention decreases of insulin/glucose ratio and HOMAR-IR. Although decreases in HOMA-β1 in the experimental group could be interpreted as a worse beta-cell function, in our opinion, this finding may result from improved insulin sensitivity and no further need of beta-cells for adjusting insulin secretion for glucose uptake in target tissues [28]. However, methodological limitations of these fasting indices of beta-cell function should be taken into account given that OGTT was not based on peripheral blood sampling, insulin levels during OGTT were not measured, and the hyperinsulinemic-euglycemic glucose clamp technique was not used.

The present results of the antidiabetic effects of *Sclerocarya birrea* are consistent with data previously reported in animal studies. In an experimental study, Wistar rats were divided into two groups and treated with a diet of oxidized palm oil and sucrose to induce hyperglycemia or a standard diet for 16 weeks; at the end of this period, animals presenting intolerance in the glucose test and insensitivity to insulin were continuously fed the hypercaloric diet along with the administration of the plant extract of *Sclerocarya birrea* (150 or 300 mg/kg) or glibenclamide (10 mg/kg) for 3 weeks. The plant extract provoked an inhibition of hyperglycemia during OGTT and a significant increase in the insulin sensitivity index [29]. These results can indicate that the plant extract could act at peripheral levels by several mechanisms, such as reducing glucose absorption from the gastrointestinal tract by inhibition of α-glycosidases and α-amylases enzymes and/or stimulating peripheral glucose utilization by a mechanism similar to that of chlorpropamide and/or metformin [29]. In another study of streptozotocin-induced diabetes in rats, there was a significant improvement in glucose tolerance in animals treated with *Sclerocarya birrea* extract and in those treated with metformin (as a reference drug [20]). The effect of *Sclerocarya birrea* similar to that of metformin could be explained by the presence of some polyphenols in the plant composition, such as epicatechin and epigallocatechin gallate, which have been shown to activate the AMP-activated protein kinase (AMPK) pathway and to inhibit glutamate dehydrogenase (GDH) activity. The hypoglycemic effect of *Sclerocarya birrea* with better control of insulin secretion and higher insulin sensitivity is still poorly understood, but other glucose-lowering pleiotropic mechanisms in line with sulfonylureas (chlorpropamide) or gallic acid have been suggested, with increased hepatic glycogen synthesis, cytosolic calcium increase with activation of calcium/calmodulin-dependent protein kinase II (CaMKII), activation of the mitogen-activated protein kinases (MAPK) and PI3K/AKT signaling pathways, and increase in glucose transporter GLUT4 [17,18,19,20,21,29,30].

Another interesting finding of the study was an improvement in FMD, which is consistent with a vasorelaxant and hypotensive effect of *Sclerocarya birrea* observed in endothelium-containing isolated aortic rings from Wistar rats [31]. This was attributed to the formation and release of endothelium-derived nitric oxide (NO) with phosphorylation of NO synthase by the polyphenols present in the plant extract.

In relation to changes in the lipid profile, significant changes in cholesterol, LDL-C, HDL-C, and triglycerides were not observed, although there was a decreasing trend of total cholesterol levels in the experimental group. However, in the study of Ngueguim et al. [29] in rats in which the plant extract was administered together with a standard diet of oxidized palm oil and sucrose for 3 weeks, there was a decrease in triglycerides, total cholesterol, LDL-C, and the atherogenic index as compared to controls, suggesting the extract of *Sclerocarya birrea* may be helpful to the prevention of diabetes complications through a reduction of dyslipidemia. We also observed a decrease of BP in the experimental group, particularly SBP, which is in agreement with a significant reduction of SBP and mean BP reported with the 300 mg/kg dose of the plant extract in the study of Ngueguim et al. [29]. In a study in normotensive rats, treatment with *Sclerocarya birrea* leaf extract induced hypotension in a dose–response manner, which was attributed to inhibition of the renin–angiotensin–aldosterone system with decreased release of the angiotensin-converting enzyme, combined with induction of the release of endothelium-derived relaxing fact/NO from endothelial cells and activation of guanylate cyclase in arterial smooth muscle and stimulation of cyclic GMP production [32].

In the present study, the use of *Sclerocarya birrea* for 90 days did not induce changes in anthropometric variables, although there was a decreasing trend in body weight. Data regarding the effect of this plant extract on the body weight and food ingestion of diabetic-induced experimental animals is inconclusive [20,32].

The analysis of inflammatory markers, including serum levels of IL-6 and E-selectin showed a significant decrease of E-selectin levels and a decreasing trend of IL-6 levels in the experimental group, pointing towards an anti-inflammatory effect of *Sclerocarya birrea*. In a study of the effect of *Sclerocarya birrea* stem-bark aqueous extract in mice, sustained and significant reductions in the fresh egg albumin-induced acute inflammation of the rat hind paw edema were observed [33]. In another study, the methanol extract of *Sclerocarya birrea* showed anti-inflammatory activity in carrageenan-, histamine- or serotonin-induced paw edema in rats through a dose-dependent inhibition of inflammatory markers such as tumor necrosis factor alpha (TNF-α) and IL-6 [34]. The anti-inflammatory effect of *Sclerocarya birrea* may interplay with the antioxidant activity reducing ROS and advanced glycation end-products [17].

The present findings, however, should be interpreted while taking into account the limitations of the study, including the exploratory nature of the trial, the reduced sample size, and the treatment period of 90 days only. Further studies assessing the use of *Sclerocarya birrea* in experimental animals and in humans using the hyperinsulinemic–euglycemic glucose clamp technique would contribute to clarifying the underlying mechanisms of the hypoglycemic effect of this natural compound in prediabetes and type 2 diabetes.

## 5. Conclusions

This exploratory clinical trial confirms the antidiabetic activity of a nutraceutical supplement based on a natural extract of *Sclerocarya birrea* in subjects with confirmed prediabetes, which is a clinically relevant finding in the prevention of type 2 diabetes. Further studies using better measurements of beta-cell function are needed to clarify the underlying mechanisms of the hypoglycemic effect of this natural compound.

## Figures and Tables

**Table 1:** Baseline characteristics of participants.

Variables	Control Group (n = 33)	Experimental Group (n = 34)
Gender		
Men	17	19
Women	16	15
Age, years, mean ± SD	31.5 ± 15.2	33.1 ± 13.2
Physical activity, %		
1 (inactive)	29.4	33.3
2 (moderately inactive)	17.6	15.2
3 (moderately active)	29.4	24.2
4 (very active)	23.5	27.3

**Table 2:** Results of changes in glucose metabolism after a 90-day use of a nutraceutical supplement of Sclerocarya birrea or placebo in subjects with prediabetes.

Variables	Pre-Intervention	Intervention Period		Significant Differences	
	Baseline	40 Days	90 Days	Within-Group *p* Value	Between-Group *p* Value
AUC of OGTT, mg·min/dL (at fasting serum glucose)					
Control group (n = 34)	4755 ± 2265	4394 ± 1879	4636 ± 1969	1.0	<0.004
Experimental group (n = 33)	5286 ± 2260	4526 ± 2543	3786 ± 1817	<0.001	
AUC of OGTT, mg·min/dL (at 0 mg/dL)					
Control group (n = 34)	17,401 ± 2284	16,892 ± 1933	17,229 ± 2152	1.0	<0.002
Experimental group (n = 33)	17,809 ± 2534	16,908 ± 2825	16,219 ± 2176	<0.001	
Glucose peak at OGTT, mg/dL					
Control group (n = 34)	179 ± 29	174 ± 25	175 ± 22	1.0	<0.011
Experimental group (n = 33)	181 ± 34	170 ± 32	161 ± 28	<0.001	
HbA1c, %					
Control group (n = 34)	5.22 ± 0.36	5.23 ± 0.40	5.22 ± 0.37	1.0	0.469
Experimental group (n = 33)	5.33 ± 0.44	5.35 ± 0.42	5.24 ± 0.43	0.543	
Fasting serum glucose, mg/dL					
Control group (n = 34)	103.4 ± 3.0	102.6 ± 4.9	103.6 ± 5.4	1.0	0.107
Experimental group (n = 33)	104.2 ± 5.0	104.3 ± 5.9	102.9 ± 5.3	0.329	
Fasting serum insulin, mU/L					
Control group (n = 34)	9.36 ± 6.55	10.80 ± 8.09	10.16 ± 6.18	1.0	<0.027
Experimental group (n = 33)	9.78 ± 6.82	7.70 ± 5.07	8.11 ± 4.46	0.148	
HOMA-IR					
Control group (n = 34)	2.40 ± 1.70	2.76 ± 2.13	2.61 ± 1.62	1.0	<0.034
Experimental group (n = 33)	2.54 ± 1.80	2.01 ± 1.38	2.08 ± 1.20	0.313	
QUICKI index					
Control group (n = 34)	0.35 ± 0.03	0.34 ± 0.03	0.34 ± 0.03	0.632	<0.034
Experimental group (n = 33)	0.35 ± 0.03	0.36 ± 0.03	0.35 ± 0.03	0.07	
HOMA-β1					
Control group (n = 34)	83.29 ± 55.81	98.49 ± 73.12	89.82 ± 50.68	1.0	<0.027
Experimental group (n = 33)	89.41 ± 65.51	70.94 ± 42.30	75.47 ± 39.21	0.438	
Insulin/glucose ratio					
Control group (n = 34)	0.09 ± 0.06	0.10 ± 0.08	0.10 ± 0.06	1.0	<0.022
Experimental group (n = 33)	0.09 ± 0.07	0.07 ± 0.05	0.08 ± 0.04	0.280	
1 h post-OGTT glucose, mg/dL					
Control group (n = 34)	161.09 ± 36.07	155.71 ± 27.56	155.00 ± 29.50	0.798	0.081
Experimental group (n = 33)	168.52 ± 39.10	154.03 ± 39.42	156.91 ± 31.11	<0.001	
2 h post-OGTT glucose, mg/dL					
Control group (n = 34)	121.35 ± 23.86	119.53 ± 20.90	124.00 ± 23.38	1.0	<0.050
Experimental group (n = 33)	125.79 ± 23.27	121.09 ± 22.01	117.12 ± 20.55	<0.028	

**Table 3:** Changes in the lipid profile after a 90-day use of a nutraceutical supplement of *Sclerocarya birrea* or placebo in subjects with prediabetes.

Variables	Pre-Intervention	Intervention Period		Significant Differences	
	Baseline	40 Days	90 Days	Within-Group *p* Value	Between-Group *p* Value
Serum cholesterol, mg/dL					
Control group (n = 34)	194 ± 34	192 ± 36	198 ± 41	1.0	0.641
Experimental group (n = 33)	191 ± 36	187 ± 38	189 ± 38	1.0	
LDL-C, mg/dL					
Control group (n = 34)	113.4 ± 25.2	114.8 ± 33.7	118.1 ± 28.1	0.610	0.662
Experimental group (n = 33)	102.8 ± 27.2	108.1 ± 30.7	111.4 ± 34.6	0.851	
HDL-C, mg/dL					
Control group (n = 34)	60.1 ± 15.1	59.0 ± 15.3	58.1 ± 13.2	0.252	0.639
Experimental group (n = 33)	59.9 ± 12.1	58.3 ± 12.1	59.1 ± 10.8	1.0	
Serum triglycerides, mg/dL					
Control group (n = 34)	94 ± 57	99 ± 61	99 ± 54	1.0	0.101
Experimental group (n = 33)	98 ± 54	87 ± 48	89 ± 42	0.531	

**Table 4:** Changes in anthropometric variables after a 90-day use of a nutraceutical supplement of *Sclerocarya birrea* or placebo in subjects with prediabetes.

Variables	Pre-Intervention	Intervention Period		Significant Differences	
	Baseline	40 Days	90 Days	Within-Group *p* Value	Between-Group *p* Value
Body weight, kg					
Control group (n = 34)	75.2 ± 18.2	75.2 ± 18.1	75.2 ± 17.9	1.0	0.469
Experimental group (n = 33)	75.2 ± 18.3	74.9 ± 18.3	74.7 ± 18.3	0.543	
Body mass index (BMI), kg/m^2^					
Control group (n = 34)	26.1 ± 5.3	26.1 ± 5.2	26.1 ± 5.2	1.0	0.261
Experimental group (n = 33)	25.9 ± 6.9	25.9 ± 6.9	25.8 ± 7.0	0.304	
Fat mass, %					
Control group (n = 34)	25.4 ± 9.3	25.3 ± 9.3	25.4 ± 9.0	1.0	0.890
Experimental group (n = 33)	29.1 ± 10.4	29.1 ± 10.6	29.3 ± 10.4	1.0	
Waist-to-hip ratio					
Control group (n = 34)	0.79 ± 0.09	0.79 ± 0.10	0.79 ± 0.10	1.0	0.928
Experimental group (n = 33)	0.79 ± 0.10	0.78 ± 0.10	0.79 ± 0.10	1.0	

**Table 5:** Changes in inflammatory biomarkers and blood pressure after 90-day use of a nutraceutical supplement of *Sclerocarya birrea* or placebo in subjects with prediabetes.

Variables	Pre-Intervention	Intervention Period		Significant Differences	
	Baseline	40 Days	90 Days	Within-Group *p* Value	Between-Group *p* Value
IL-6, pg/mL					
Control group (n = 34)	4.37 ± 1.37	4.43 ± 1.25	4.36 ± 1.41	1.0	0.284
Experimental group (n = 33)	4.34 ± 1.79	4.17 ± 1.71	4.08 ± 1.41	0.159	
E-selectin, ng/mL					
Control group (n = 34)	16.62 ± 1.53	16.59 ± 1.45	16.23 ± 0.96	0.878	0.001
Experimental group (n = 33)	17.59 ± 1.11	16.52 ± 0.99	15.70 ± 1.06	0.001	
Blood pressure (BP)					
Systolic BP, mmHg					
Control group (n = 34)	120 ± 18	119 ± 17	120 ± 16	1.0	0.259
Experimental group (n = 33)	125 ± 18	122 ± 18	119 ± 17	0.001	
Diastolic BP, mmHg					
Control group (n = 34)	76 ± 11	78 ± 10	76 ± 9	1.0	0.132
Experimental group (n = 33)	78 ± 10	76 ± 11	76 ± 12	0.265	

## Data Availability

Study data are available from the corresponding author (F.J.L.-R.) upon request.
